# Comparative risk of hospitalized infection between biological agents in rheumatoid arthritis patients: A multicenter retrospective cohort study in Japan

**DOI:** 10.1371/journal.pone.0179179

**Published:** 2017-06-08

**Authors:** Shunsuke Mori, Tamami Yoshitama, Toshihiko Hidaka, Fumikazu Sakai, Mizue Hasegawa, Yayoi Hashiba, Eiichi Suematsu, Hiroshi Tatsukawa, Akinari Mizokami, Shigeru Yoshizawa, Naoyuki Hirakata, Yukitaka Ueki

**Affiliations:** 1 Department of Rheumatology, Clinical Research Center for Rheumatic Diseases, National Hospital Organization (NHO) Kumamoto Saishunsou National Hospital, Kohshi, Kumamoto, Japan; 2 Yoshitama Clinic for Rheumatic Diseases, Kirishima, Kagoshima, Japan; 3 Institute of Rheumatology, Zenjinkai Shimin-no-Mori Hospital, Miyazaki, Japan; 4 Department of Diagnostic Radiology, Saitama Medical University International Medical Center, Hidaka, Saitama, Japan; 5 Department of Respiratory Medicine, Tokyo Women’s Medical University Yachiyo Medical Center, Yachiyo, Chiba; 6 Department of Internal Medicine and Rheumatology, Clinical Research Institute, NHO Kyushu Medical Center, Fukuoka, Japan; 7 Department of Rheumatology, Oita Red Cross Hospital, Oita, Japan; 8 Department of Rheumatology, Japan Community Healthcare Organization (JCHO) Isahaya General Hospital, Isahaya, Nagasaki, Japan; 9 Department of Rheumatology, NHO Fukuoka National Hospital, Fukuoka, Japan; 10 Rheumatic and Collagen Disease Center, Sasebo Chuo Hospital, Sasebo, Nagasaki, Japan; Keio University, JAPAN

## Abstract

**Objective:**

Knowing the risk of hospitalized infection associated with individual biological agents is an important factor in selecting the best treatment option for patients with rheumatoid arthritis (RA). This study examined the comparative risk of hospitalized infection between biological agents in a routine care setting.

**Methods:**

We used data for all RA patients who had first begun biological therapy at rheumatology divisions of participating community hospitals in Japan between January 2009 and December 2014. New treatment episodes with etanercept, infliximab, adalimumab, abatacept, or tocilizumab were included. Patients were allowed to contribute multiple treatment episodes with different biological agents. Incidence rates (IRs) of hospitalized infection during the first year of follow-up were examined. Cox regression analysis was used to calculate hazard ratios (HRs) for overall hospitalized infection and for pulmonary hospitalized infection, adjusting for possible confounders.

**Results:**

A total of 1596 new treatment episodes were identified. The incidence of overall hospitalized infection during the first year was 86 with 1239 person-years (PYs), yielding a crude IR of 6.9 per 100 PYs (95% confidence interval [CI], 5.6–8.6). After correction for confounders, no significant difference in risk of hospitalized infection was observed between treatment groups: adjusted HRs (95% CI) were 1.54 (0.78–3.04) for infliximab, 1.72 (0.88–3.34) for adalimumab, 1.11 (0.55–2.21) for abatacept, and 1.02 (0.55–1.87) for tocilizumab compared with etanercept. Patient-specific factors such as age, RA functional class, body mass index (BMI), prednisolone use, and chronic lung disease contributed more to the risk of hospitalized infection than specific biological agents. The incidence of pulmonary hospitalized infection was 50 and a crude IR of 4.0 per 100 PYs (95% CI, 3.1–5.3). After adjustment for confounders, adalimumab had a significantly higher HR for pulmonary hospitalized infection compared with tocilizumab: an adjusted HR (95% CI) was 4.43 (1.72–11.37) for adalimumab. BMI, prednisolone use, diabetes mellitus, and chronic lung disease were also significant factors associated with the risk of pulmonary hospitalized infection.

**Conclusions:**

The magnitude of the risk of overall hospitalized infection was not determined by the type of biological agents, and patient-specific risk factors had more impact on the risk of hospitalized infection. For pulmonary hospitalized infections, the use of adalimumab was significantly associated with a greater risk of this complication than tocilizumab use.

## Introduction

Over the past decade, clinical and social outcomes of rheumatoid arthritis (RA) patients have dramatically improved with aggressive intervention with methotrexate (MTX) early in the disease course, coupled with the widespread use of biological agents that target specific components in the immune system. The biological agents have shown great ability to relieve RA symptoms, slow disease progression, prevent joint damage, and improve physical function and quality of life [1–4]. Since its first approval for RA in 2003 in Japan, biological treatment has gained popularity as a potent therapeutic option for RA patients who have experienced failure in MTX therapy. Anti-tumor necrosis factor-α (TNFα) antibodies (infliximab, adalimumab, golimumab, and certolizumab), a soluble TNF receptor (etanercept), an anti-interleukin-6 receptor antibody (tocilizumab), and an inhibitor of T-cell costimulatory signaling (abatacept) are mainly used in biological therapy for RA in Japan.

Serious infection is one of the most important concerns for patients with RA who are treated with biological agents. Using data from randomized controlled trials (RCTs), several groups conducted a systemic review and meta-analysis of this risk, and their evidence is conflicting for an increased risk of serious infection at recommended doses of biological agents compared with placebo or non-biological antirheumatic drugs [5–14]. Considering the nature of the RCT design (i.e., relatively short duration of follow-up, selected patient populations [limited to patients without significant comorbidity and disability], and unequal exposure to active and control therapies for ethical reasons), meta-analyses of data from these trials may not allow an assessment of safety profiles for real-world RA patients. In this context, large observational studies, clinical registries, and health care databases have provided useful data on the true risk of these therapies in clinical practice [15–17]. However, these studies have also shown conflicting results regarding the safety of biological agents, with some studies detecting a strong association of serious infection with the use of biological agents and others identifying a small or no increase in the risk [18–34].

Given different mechanisms of action between specific drugs and drug classes, there is a possibility that the risk of serious infection may differ between specific biological agents. For selection of the best treatment option for RA patients, we need to carefully compare the risk of serious infection between currently available biological agents. Using data from clinical registries and health care databases, several studies addressed this critical issue, but data used were obtained from those RA patients who had received biological therapies from the mid-2000s up to 2011 [31, 35–42]. Increasing physician awareness of the infectious risk and improvement in the management of RA patients who are scheduled for and receiving biological therapies can change the incidence and risk of serious infection associated with biological therapies.

To assess the comparative risk of hospitalized infection between biological agents based on new real-world data, we conducted a multicenter retrospective cohort study with a follow-up time of up to 1 year, using data from 2009–2014 for all RA patients who had first received biological therapy at eight community hospitals in Japan (the SARABA study: the SAfety profile of RA patients receiving Biological Agents study).

## Methods

### Patients and study design

The SARABA study was designed to compare the risk of hospitalized infection between biological agents used to treat RA in a routine care setting. For this study, we used databases including all RA patients who had first begun biological therapy at rheumatology divisions of the following community hospitals in Japan between January 2009 and December 2014: NHO Kumamoto Saishunsou National Hospital, Yoshitama Clinic for Rheumatic Diseases, Zenjinkai Shimin-no-Mori Hospital, NHO Kyushu Medical Center, Oita Red Cross Hospital, JCHO Isahaya General Hospital, NHO Fukuoka Hospital, and Sasebo Chuo Hospital. All patients were required to fulfill the 1987 American College of Rheumatology (ACR) criteria or the 2010 ACR/European League Against Rheumatism (EULAR) criteria for diagnosis of RA [43, 44]. Patients who had received biological therapy at any time before this period were excluded from the study. The treatment effect of the following five biological agents on the incidence of hospitalized infection were compared in this study: infliximab, adalimumab, etanercept, abatacept, and tocilizumab. Because certolizumab and golimumab were used for only a small number of patients, statistical analysis could not be performed on these agents independently. Rituximab is currently not approved for treatment of RA in Japan.

A treatment episode was defined as the initiation of a new course of biological therapy. An outcome was the first occurrence of hospitalized infection during the follow-up periods. Index date (start date of follow-up) was defined as the starting time of a new treatment episode. Follow-up ended at the earliest date (the endpoint data) of the following: a first hospitalization that resulted from infection, 12 months after the initiation of a biological agent, the time a currently used biological agent was discontinued, loss to follow-up, or death. Patients were considered to have discontinued treatment with each biological agent if they did not take that specific agent after the recommended dosing interval had passed, regardless of whether they subsequently switched to another biological agent. Days of exposure to each biological agent were assigned as the number of days between the index date and the endpoint date. If the drug was discontinued, patients were considered to have been exposed to that specific biological agent until the end of the recommended dosing interval. Eligible patients were allowed to contribute multiple episodes of treatment with a biological agent if they started a new course of treatment with a different biological agent.

### Data collection

Demographic characteristics (age and sex), RA-related features (RA duration and Steinbrocker’s RA stage and class), body mass index (BMI), and smoking history at baseline were examined within 2 weeks before the index date for each episode of biological therapy. Steinbrocker’s RA class was used as a baseline index for patient physical disability instead of the health assessment questionnaire [HAQ] [31, 40]. Comorbid diseases (chronic kidney disease [CKD], diabetes mellitus, and chronic lung disease) were also examined at the same time. CKD was defined as absolute estimated glomerular filtration rate (eGFR) <60 ml/min lasting for 3 months or longer [45]. Diabetes was defined as fasting blood glucose levels of 126 mg/dl or higher, serum HbAc1 higher than 6.0%, or the use of diabetes medications. Chronic lung disease included interstitial lung disease, bronchiolitis, bronchiectasis, and pulmonary emphysema, which were diagnosed according to abnormal findings on high-resolution computed tomography (HRCT). Patients’ chest HRCT images were reviewed in random order and independently by two experts in thoracic radiology and diagnostic imaging (FS and MH). Both observers were blinded to the patients’ clinical status. Final diagnosis was determined by consensus if there was a disagreement between their interpretations. Concurrent use of MTX and prednisolone during biological therapy as well as previous use of biological agents prior to each treatment episode, if applicable, were also extracted from the databases.

All data at baseline and during follow-up were extracted from each division’s database by site investigators and data sheets were submitted to the Data Center for the SARABA Study at the Clinical Research Center for Rheumatic Diseases of NHO Kumamoto Saishunsou National Hospital. The site investigators have confirmed that all treatment episodes with biological agents were captured from the registries of their divisions and that the data on comorbidities were accurately collected according to the above-mentioned definitions. To reduce inter-center or inter-physician differences, the Data Center reviewed all data submitted by the site investigators, especially focusing on fulfillment of the inclusion and exclusion criteria at enrollment, data quality at baseline, and protocol adherence during follow-up. If verification of the accuracy in these points was required, the Data Center sent an inquiry letter to the site investigators, and questions were resolved by consensus of the investigator team.

### Hospitalized infection

Type, number, and hospitalization days for the infectious disease during the follow-up period, together with mortality associated with these adverse events, were examined. Infectious disease diagnosis and hospital admission decisions were made by the treating physicians at the participating hospitals based on a comprehensive evaluation of physical findings, laboratory findings, and radiological examinations. Validity of physician-reported hospitalized infection was confirmed by reviewing patients’ medical records and discharge summaries (including International Classification of Diseases Version 10 [ICD-10] codes) by site investigators. The Data Center also reviewed case report forms sent by site investigators and confirmed anatomic sites of each infectious episode, microorganism data, and hospitalization days. Hospitalized infections were categorized by anatomic sites and organisms based on ICD-10 codes listed as the primary reason for hospitalization. For patients having two or more diagnoses of infectious diseases during a single hospitalization, we selected the infectious disease that had caused most severe patient conditions and had therefore used the most medical resources. Patients who were hospitalized to be examined for infection were not included. The severity of hospitalized infection was determined by the number of hospitalization days and the requirement for intravenous antibiotic treatment if it was a bacterial infection. In this cohort, a median duration (interquartile range) of hospitalization was 14 days (8–28 days), and all cases of bacterial infection received intravenous antibiotics. Mortality was determined by identifying patients who died within 30 days after hospitalization.

### Ethics approval

This study was conducted in accordance with the principles of the Declaration of Helsinki (2008). The protocol of this study also meets the requirements of the Ethical Guidelines for Medical and Health Research Involving Human Subjects, Japan (2014) and has been approved by the Human Research Ethics Committees of NHO Kumamoto Saishunsou National Hospital. This study is a retrospective cohort study, not an intervention study to human subjects, and the data were analyzed anonymously. Therefore, patient informed consent to participate was not required. This study is registered with the University Hospital Medical Information Network-Clinical Trials Registry (registry number: UMIN000016412).

### Statistical analysis

To compare demographic and clinical characteristics of patients at baseline across the treatment groups with different biological agents, we calculated the mean, standard deviation (SD), and number (%). Continuous variables were assessed using analysis of variance (ANOVA) with a post-hoc Dunnett’s T3 test. The chi-square test or the Fisher’s exact probability test was used for categorical variables.

The incidence rates (IRs) of hospitalized infection with 95% confidence intervals (CIs) were calculated by dividing the number of incidence cases by the number of corresponding biologic-exposed person-years (PYs) for each treatment group. Cumulative incidence of hospitalized infection during the 12-month follow-up period for each treatment group was computed from life tables using the Kaplan-Meier approach.

Cox regression analysis was used to calculate hazard ratios (HRs) for overall hospitalized infection or pulmonary hospitalized infection associated with exposure to individual biological agents, adjusting for confounders. Possible confounders that were used in the Cox regression analysis included age, sex, RA duration, RA stage III/IV, RA class 3/4, BMI, smoking history, previous use of biological agents, concurrent use of MTX, concurrent use of prednisolone, and comorbid diseases (CKD, diabetes mellitus, chronic lung disease). Variables were considered to be true confounders if estimates for the main effect (effects of exposure to biological agents) changed by more than 10% when adding each variable sequentially to a Cox regression model using a forward selection procedure [37, 46, 47]. All variables identified as the true confounders were then included in Cox regression analyses to provide estimates of the treatment effect on overall hospitalized infection or pulmonary hospitalized infection, adjusting for these true confounders. Risk differences between biological treatments are presented as adjusted HRs with 95% CI.

The proportional hazards assumption was checked using log-minus-log (LML) plots of log cumulative hazard curve function and scaled Schoenfeld residual (SR) plots for exposure variables over time. Parallel, non-crossing LML plots and horizontal SR plots indicated that there was no significant violation of the proportional hazards assumption for any of the exposure variables during the follow-up period.

For all tests, probability values (*p* values) < 0.01 were considered to indicate statistical significance. All calculations were performed using either Excel Statistical Analysis 2010 (SSRI Co., Ltd., Tokyo, Japan) or PASW Statistics version 22 (SPSS Japan Inc., Tokyo, Japan).

## Results

### Demographic and clinical characteristics of RA patients at the start of a new course of biological therapy

A total of 1596 treatment episodes with biological agents for RA patients were identified. Among these episodes, 413 (25.9%) were with etanercept, 335 (21.0%) with infliximab, 264 (16.5%) with adalimumab, 189 (11.8%) with abatacept, and 395 (24.7%) with tocilizumab (Table 1).

**Table 1:** Demographic and clinical characteristics of RA patients at the start of a new course of biological therapy.

	All episodes (n = 1596)	ETN (n = 413)	IFX (n = 335)	ADA (n = 264)	ABT (n = 189)	TCZ (n = 395)
Age, years, mean (SD)*	60.9 (14.2)	62.7 (13.3)	55.7 (12.2)	57.3 (13.7)	66.3 (12.9)	66.3 (12.9)
Female sex, patient number (%)	1237 (77.5)	317 (76.8)	262 (78.2)	209 (79.2)	142 (75.1)	307 (77.7)
RA duration, years, mean (SD) ^†^	7.8 (9.5)	8.5 (10.8)	6.3 (7.9)	7.9 (10.3)	9.1 (9.7)	7.8 (8.6)
Class 3/4, patient number (%) ^‡^	245 (15.4)	80 (19.4)	37 (11.0)	41 (15.5)	37 (19.6)	50 (12.7)
Stage III/IV, patient number (%) ^§^	688 (43.1)	186 (45.0)	126 (37.6)	106 (40.2)	94 (49.7)	176 (44.6)
Body mass index < 18.5, patient number (%)	205 (12.8)	57 (13.8)	44 (13.1)	27 (10.2)	28 (14.8)	49 (12.4)
First biological therapy, patient number (%) ^¶^	1125 (70.5)	341 (82.6)	281 (83.9)	198 (75)	98 (51.9)	207 (52.4)
Concurrent RA medications						
MTX use, patient number (%) **	1125 (70.5)	222 (53.8)	335 (100)	223 (84.5)	114 (60.3)	231 (58.5)
MTX dose, mg/week, mean (SD) ^††^	8.9 (2.8)	8.9 (2.8)	8.9 (2.5)	8.2 (2.4)	9.7 (3.3)	8.8 (3.1)
Prednisolone use, patient number (%) ^‡‡^	797 (49.9)	211 (51.1)	131 (39.1)	129 (48.9)	109 (57.7)	217 (54.9)
Prednisolone dose, mg/day, mean (SD) ^§§^	5.8 (3.7)	6.5 (4.0)	5.1 (2.7)	5.9 (4.0)	5.6 (3.6)	5.6 (3.7)
Comorbidities						
Chronic kidney disease, patient number (%) ^¶¶^	208 (13.0)	76 (18.4)	18 (5.4)	23 (8.7)	31 (16.4))	60 (15.2)
NIDDM, patient number (%) ***	200 (12.5)	66 (16.0)	26 (7.8)	28 (10.6)	24 (12.7)	56 (14.2)
Chronic lung disease, patient numbers (%) ^†††^	397 (24.9)	126 (30.5)	57 (17.0)	44 (16.7)	72 (38.1)	98 (24.8)
Smoking (≥10 pack-years), patient numbers (%)	355 (22.2)	84 (20.3)	81 (24.2)	56 (21.2)	48 (25.4)	86 (21.8)

RA, rheumatoid arthritis; ETN, etanercept; IFX, infliximab; ADA, adalimumab; ABT, abatacept; TCZ, tocilizumab; MTX methotrexate; NIDDM, non-insulin-dependent diabetes mellitus; HRCT, high-resolution computed tomography; ANOVA, analysis of variance; SD, standard deviation. Differences between treatment groups were assessed using ANOVA with a post-hoc Dunnett’s T3 test, the chi-square test, or Fisher’s exact probability test.

*p < 0.001 for comparisons of IFX vs. ETN, IFX vs. ABT, IFX vs. TCZ, ADA vs. ETN, ADA vs. ABT, and ADA vs. TCZ; *p* = 0.018 for ABT vs. TCZ

^†^*p* = 0.007 for IFX vs. ABT; *p* = 0.013 for IFX vs. ETN

^‡^*p* = 0.002 for IFX vs. ETN; *p* = 0.009 for IFX vs. ABT; *p* = 0.011 for ETN vs. TCZ, *p* = 0.034 for ABT vs. TCZ

^§^*p* = 0.008 for IFX vs. ABT; *p* = 0.040 for ADA vs. ABT; *p* = 0.044 for IFX vs. ETN

^¶^*p* < 0.001 for ETN vs. ABT, ETN vs. TCZ, IFX vs. ABT, IFX vs. TCZ, ADA vs. ABT, and ADA vs. TCZ; *p* = 0.008 for IFX vs. ADA; *p* = 0.019 for ETN vs. ADA

**p < 0.001 for IFX vs. ETN, IFX vs. ADA, IFX vs. ABT, IFX vs. TCZ, ADA vs. ETN, ADA vs. ABT, and ADA vs. TCZ

^††^*p* < 0.001 for ADA vs. ABT; *p* = 0.006 for IFX vs. ADA; *p* = 0.036 for IFX vs. ABT

^‡‡^*p* < 0.001 for IFX vs. ABT, IFX vs. TCZ, and IFX vs. ETN; *p* = 0.020 for IFX vs. ADA

^§§^*p* = 0.006 for IFX vs. ETN

^¶¶^*p* < 0.001 for IFX vs. ETN, IFX vs. ABT, and IFX vs. TCZ, and ADA vs. ETN; *p* = 0.016 for ADA vs. TCZ; *p* = 0.018 for ADA vs. ABT

***p = 0.001 for IFX vs. ETN; *p* = 0.007 for IFX vs. TCZ

^†††^Including interstitial lung disease, bronchiolitis, bronchiectasis, and pulmonary emphysema. The diagnosis was made based on HRCT findings. *p* < 0.001 for IFX vs. ETN, IFX vs. ABT, ETN vs. ADA, and ADA vs. ABT; *p* = 0.001 for ABT vs. TCZ; *p* = 0.011 for IFX vs. TCZ; *p* = 0.015 for ADA vs. TCZ

The mean age of the patients was 60.9 years, and most patients were female (77.5%). Patients in the treatment groups with the antibody type of anti-TNFα agents (infliximab and adalimumab) were significantly younger than those in the other treatment groups (etanercept, abatacept, and tocilizumab; *p* < 0.001). There was no significant difference in sex, BMI <18.5, or smoking history between the five treatment groups. RA duration was shorter in patients treated with infliximab compared with those receiving etanercept and abatacept. For patients with functionally severe RA (classes 3 and 4) and those with radiologically advanced RA (stages III and IV), infliximab was less often prescribed compared with etanercept and abatacept. Anti-TNFα agents (etanercept, infliximab, and adalimumab) were more often used as the first biological agent compared with abatacept and tocilizumab (*p* < 0.001). The concurrent use of MTX was more frequently observed in treatment episodes with the antibody type of anti-TNFα agents compared with those with etanercept, abatacept, and tocilizumab (*p* < 0.001), and MTX was less commonly used in tocilizumab therapy compared with the other biological therapies (*p* < 0.001). Significantly lower doses of MTX were used for patients receiving adalimumab compared with those treated with abatacept (*p* < 0.001) or infliximab (*p* = 0.006). Prednisolone was less commonly used for patients receiving infliximab compared with abatacept, tocilizumab, and etanercept (*p* < 0.001). Median doses of prednisolone at baseline were significantly lower in infliximab therapy compared with etanercept (*p* = 0.006). The antibody type of anti-TNFα agents, especially infliximab, were also less likely to be prescribed for CKD patients than other types of biological agents (*p* < 0.001).

### Occurrence of hospitalized infection by infection sites

The type and number of hospitalized infections across treatment groups during the first year of follow-up are shown in Table 2. Pulmonary infections, especially bacterial pneumonia, occurred most frequently in all treatment groups. Among 86 patients who had developed hospitalized infections, 7 (8.1%) eventually died during or within 30 days after hospitalization.

**Table 2:** Type, number, and mortality associated with hospitalized infections during the first year of follow-up to biological therapy for RA.

	All episodes n = 1596	ETN n = 413	IFX n = 335	ADA n = 264	ABT n = 189	TCZ n = 395
Pulmonary infection	50	16	8	12	7	7
Bacterial pneumonia	31	13	2	7	3	6
Empyema	1	1	0	0	0	0
Viral pneumonia	2	1	1	0	0	0
Bronchiolitis	1	0	0	1	0	0
NTM disease	3	0	1	0	1	1
Pulmonary mycosis	4	0	0	1	3	0
Tuberculous pleurisy	1	0	0	1	0	0
PCP	7	1	4	2	0	0
Gastrointestinal infection	6	2	0	0	1	3
Cholecystitis	2	0	0	0	1	1
Diverticulitis	1	0	0	0	0	1
Infectious enteritis	3	2	0	0	0	1
Skin and soft tissue	8	2	1	0	1	4
Cellulitis	6	2	1	0	1	2
Decubitus infection	1	0	0	0	0	1
Perirectal abscess	1	0	0	0	0	1
Urinary tract infection	8	1	1	2	2	2
Pyelonephritis	8	1	1	2	2	2
Musculoskeletal Infection	10	2	3	1	1	3
Iliopsoas abscess	1	0	0	0	0	1
Masseter abscess	1	1	0	0	0	0
Osteomyelitis	2	0	0	0	1	1
Pyogenic arthritis	4	1	3	0	0	0
Prosthetic infection	1	0	0	1	0	0
Pyogenic spondylitis	1	0	0	0	0	1
Other	3	1	2	0	0	0
Tonsillitis	3	1	2	0	0	0
Sepsis	1	1	0	0	0	0
Total (%)	86 (5.4)	25 (6.1)	15 (4.5)	15 (5.7)	12 (6.3)	19 (4.8)
Mortality (%)	7 (8.1)	4 (16.0)	0	1 (6.7)	1 (8.3)	1 (5.3)

Data are expressed as number of infections. Mortality is shown as number of deaths (%). RA, rheumatoid arthritis; ETN, etanercept; IFX, infliximab; ADA, adalimumab; ABT, abatacept; TCZ, tocilizumab; NTM, nontuberculous mycobacteria; PCP, *Pneumocystis jirovecii* pneumonia

### Incidence and risk of hospitalized infection during the first year in RA patients receiving biological agents

**Fig 1:**
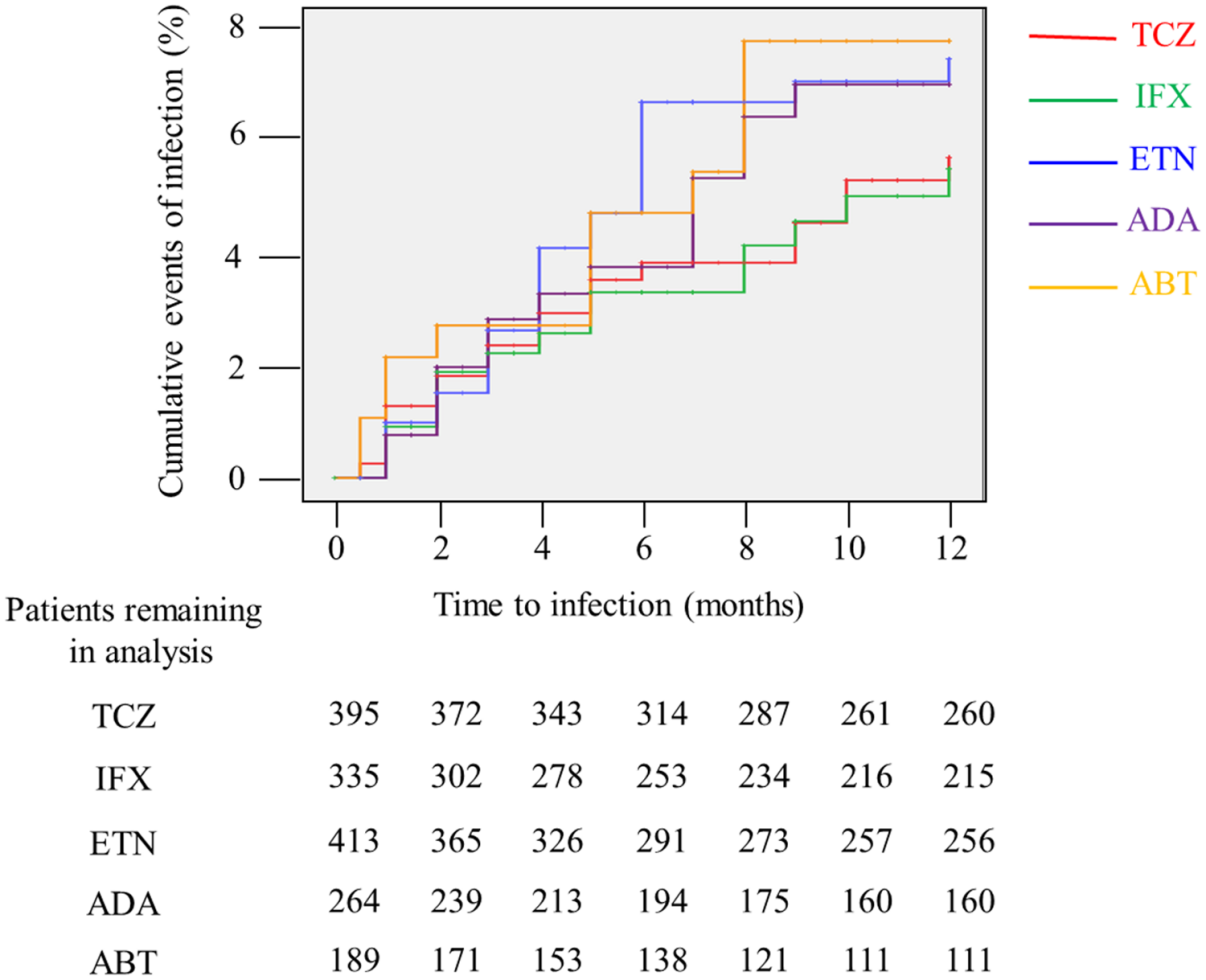
Cumulative incidence of hospitalized infection during the first year of follow-up, according to biological agent. Numbers below this figure represent the number of patients remaining in the analysis. TCZ, tocilizumab; IFX, infliximab; ETN, etanercept; ADA, adalimumab; ABT, abatacept.

The total incidence of hospitalized infection during the first year of follow-up was 86 with 1239 PYs of exposure to biological agents, yielding a crude IR of 6.9 (95% CI, 5.6–8.6) per 100 PYs (Table 3). Crude IRs across different biological agents ranged from 5.7 per 100 PYs (infliximab) to 8.4 per 100 PYs (abatacept). The cumulative incidence of hospitalized infection for each of the five biological agents during the first year of follow-up is shown in Fig 1. The plot suggested that there was little difference in the incidence of hospitalized infection based on the type of biological agent used. Crude HRs for individual biological agents were similar. By sequentially adding each covariate to the Cox regression model, we identified age, class 3/4, BMI <18.5, concurrent prednisolone use, and chronic lung disease as the true confounders. After adjustment of HRs for these cofounders, we found that there was no significant difference in risk between treatment groups: the adjusted HR (95% CI) was 1.54 (0.78–3.04) for infliximab (*p* = 0.22), 1.72 (0.88–3.34) for adalimumab (*p* = 0.11), 1.11 (0.55–2.21) for abatacept (*p* = 0.78), and 1.02 (0.55–1.87) for tocilizumab (*p* = 0.96) compared with etanercept. In the final Cox regression mode, the true confounders, especially age, class 3/4, BMI <18.5, prednisolone use (≥7.5 mg/day), and chronic lung disease, were strongly and significantly associated with the increased risk of hospitalized infection (*p* < 0.01). The effect of prednisolone use on the risk of hospitalized infection was dose-dependent (Table 3).

**Table 3:** Comparison of the risk of hospitalized infection during the first year of follow-up between treatment groups of RA patients.

	No. of events	PYs	Crude IR per 100 PYs (95% CI)	Crude HR (95% CI)	*p* value	Adjusted HR* (95% CI)	*p* value
Total (n = 1596)	86	1239	6.9 (5.6–8.6)	-	-	-	-
Etanercept (n = 413)	25	312	8.0 (5.4–11.9)	1.39 (0.73–2.63)	0.32	reference	-
Infliximab (n = 335)	15	262	5.7 (3.5–9.5)	reference	-	1.54 (0.78–3.04)	0.22
Adalimumab (n = 264)	15	202	7.4 (4.5–12.3)	1.29 (0.63–2.63)	0.49	1.72 (0.88–3.34)	0.11
Abatacept (n = 189)	12	143	8.4 (4.8–14.8)	1.45 (0.68–3.09)	0.34	1.11 (0.55–2.21)	0.78
Tocilizumab (n = 395)	19	319	6.0 (3.8–9.4)	1.04 (0.53–2.05)	0.91	1.02 (0.55–1.87)	0.96
Age	-	-	-	-	-	1.04 (1.02–1.06)	<0.001
Class 3/4	-	-	-	-	-	1.92 (1.20–3.09)	0.007
Body mass index < 18.5	-	-	-	-	-	2.55 (1.57–4.14)	<0.001
Prednisolone use^†^							
≥ 7.5 mg/day	-	-	-	-	-	3.56 (2.15–5.88)	<0.001
≥ 5 and < 7.5 mg/day	-	-	-	-	-	1.88 (1.10–3.22)	0.022
HRCT-proven chronic lung disease	-	-	-	-	-	1.85 (1.17–2.92)	0.008

*Cox regression analysis was performed to compare treatment effect of each biological agent on hospitalized infection, after adjustment for possible confounders. The possible confounders included age, sex, BMI, smoking history, RA duration, RA stage III/IV, RA class 3/4, previous use of biological agents, concurrent use of MTX, concurrent use of prednisolone, and comorbid diseases (chronic kidney disease, diabetes mellitus, and chronic lung disease). All confounders listed in the table were factors identified as the true confounders and included in the final Cox model.

^†^Referent to no use of prednisolone.

RA, rheumatoid arthritis; PY, person-year; IR, incidence rate; HR, hazard ratio; 95% CI, 95% confidence interval; HRCT, high-resolution computed tomography

### Incidence and risk of pulmonary hospitalized infection during the first year in RA patients receiving biological agents

The total incidence of pulmonary hospitalized infection during the first year of follow-up was 50, with 1239 PYs of exposure to biological agents (Table 4). A crude IR in treatment episodes with biological therapy was 4.0 (95% CI, 3.1–5.3) per 100 PYs, ranging from 2.2 per 100 PYs (tocilizumab) to 5.9 per 100 PYs (adalimumab). By sequentially adding each covariate to the Cox regression model, we identified age, BMI, concurrent prednisolone use, diabetes mellitus, and chronic lung disease as the true confounders. After adjustment for the true confounders, the risk of pulmonary hospitalized infection was significantly higher in the adalimumab group compared with tocilizumab; the adjusted HR (95% CI) was 4.43 (1.72–11.37) for adalimumab (*p* = 0.002). In the final Cox regression mode, the risk of pulmonary hospitalized infection was significantly greater in RA patients with the true confounders, in particular BMI <18.5, prednisolone use (≥7.5 mg/day), diabetes mellitus, and chronic lung disease (*p* < 0.01), compared with those without these confounders. The risk of pulmonary hospitalized infection was not significantly increased in patients receiving low doses of prednisolone (Table 4).

**Table 4:** Comparison of the risk of pulmonary hospitalized infection during the first year of follow-up between treatment groups of RA patients.

	No. of events	PY	Crude IR per 100 PY (95% CI)	Crude HR (95% CI)	*p* value	Adjusted HR* (95% CI)	*p* value
Total (n = 1596)	50	1239	4.0 (3.1–5.3)	-	-	-	-
Etanercept (n = 413)	16	312	5.1 (3.1–8.4)	2.33 (0.96–5.67)	0.06	1.65 (0.67–4.09)	0.28
Infliximab (n = 335)	8	262	3.1 (1.5–6.1)	1.40 (0.51–3.85)	0.52	2.07 (0.75–5.76)	0.16
Adalimumab (n = 264)	12	202	5.9 (3.4–10.5)	2.67 (1.06–6.85)	0.002	4.43 (1.72–11.37)	0.002
Abatacept (n = 189)	7	143	4.9 (1.9–9.3)	2.21 (0.78–6.32)	0.14	1.63 (0.56–4.72)	0.37
Tocilizumab (n = 395)	7	319	2.2 (1.0–4.6)	reference	-	reference	-
Age	-	-	-	-	-	1.03 (1.01–1.06)	0.018
Body mass index < 18.5	-	-	-	-	-	2.90 (1.56–5.39)	0.001
Prednisolone use^†^							
≥ 7.5 mg/day	-	-	-	-	-	2.49 (1.36–4.54)	0.003
NIDDM	-	-	-	-	-	2.45 (1.30–4.61)	0.005
HRCT-proven chronic lung disease	-	-	-	-	-	3.61 (1.94–6.73)	< 0.001

*Cox regression analysis was performed to compare treatment effect of each biological agent on pulmonary hospitalized infection, after adjustment for possible confounders. The possible confounders included age, sex, BMI, smoking history, RA duration, RA stage III/IV, RA class 3/4, previous use of biological agents, concurrent use of MTX, concurrent use of prednisolone, and comorbid diseases (chronic kidney disease, diabetes mellitus, and chronic lung disease). All confounders listed in the table are factors identified as the true confounders and included in the final Cox model.

^†^Referent to no use of prednisolone.

RA, rheumatoid arthritis; PY, person-year; IR, incidence rate; HR, hazard ratio; 95% CI, 95% confidence interval; NIDDM, non-insulin-dependent diabetes mellitus; HRCT, high-resolution computed tomography

## Discussion

In this retrospective cohort study using 2009–2014 data for new treatment episodes with the five biological agents in the eight participating institutions, we found that there was no significant difference in adjusted HRs of overall hospitalized infection during the first year of follow-up of the RA patients receiving etanercept, infliximab, adalimumab, abatacept, or tocilizumab. Patient-specific factors contributed more to the increased risk of hospitalized infection than did specific biological agents. Pulmonary infection was the most frequent infection requiring hospitalization during biological therapy. After adjustment for confounders, the use of adalimumab was significantly associated with a greater risk of pulmonary hospitalized infection compared with tocilizumab.

There are studies that indirectly compared the risk of serious infection between biological agents through meta-analyses of data from RCTs and open-label extension studies [6–12]. Several studies concluded that some biological agents were associated with a higher risk of serious infection compared to others [9, 12], but other studies showed that there was no significant increase in the risk of serious infection during biological therapies at the recommended doses [6–8, 10, 11]. Differences in control arms (placebo, MTX, or placebo and other concurrent non-biological disease modifying antirheumatic drugs [DMARDs]) and patient selection (biologic-naïve RA, MTX-naïve RA, or RA refractory to these DMARDs) included in these studies may hamper precise comparisons between biological agents in this type of meta-analysis.

Through a direct comparison using data from national registries and health care databases, several studies reported the comparative safety of various biological agents in RA patients, with a focus on serious infection [19, 31, 35–42]. Using a database from a large US healthcare organization from 2005–2009, Curtis et al. showed that users of etanercept, adalimumab, or abatacept had lower rates of hospitalized infection compared with users of infliximab [35]. Using data from the DREAM registry up to 2011, van Dartel et al. indicated that the risk of serious infection in RA patients treated with infliximab or adalimumab was significantly higher compared with etanercept [37]. Similar results were reported by Atzeni et al. with 2008–2011 data from the GISEA registry [36]. Using 2005–2007 data from the Japanese REAL registry, however, Sakai et al. revealed that infliximab had no significantly higher association with hospitalized infection compared with etanercept [31]. Using 2006–2011 data from the same registry, they also showed that the risk of serious infection was comparable between RA patients receiving tocilizumab and those treated with anti-TNFα agents [40]. Using 2001–2005 data from the British Society for Rheumatology Biologics Registry, Dixon et al. also showed that there was no significant difference in adjusted incidence rate ratios (IRRs) of serious infection among each group treated with infliximab, adalimumab, or etanercept [19].

Several direct-comparison studies targeted particular patient groups: previous users of biological agents, patients of advanced age, and patients with a history of previous infection. Using Medicare data from 2006–2011 for RA patients who had previous exposure to a biological agent and were then switched to another agent, Yun et al. showed that exposure to etanercept, infliximab, or rituximab was associated with an increased risk of hospitalized infection during the first year compared with exposure to abatacept, while there was no significant increase in the risk for adalimumab, certolizumab, golimumab, or tocilizumab compared to abatacept [41]. Using a large US claims database from 2004–2010, Johnston et al. showed different results: they reported that in RA patients switching from anti-TNFα agents to another biological agent, the risk of severe infection was higher for infliximab, abatacept, adalimumab, and etanercept compared with rituximab [42]. Using 1998–2011 data from the US Veterans Health Administration, Curtis et al. showed that in the older, predominantly male RA cohort, and compared to etanercept, the risk of hospitalized bacterial infections was not different for abatacept or rituximab, while it was higher for infliximab [38]. Using 2006–2010 Medicare data for RA patients, Yun et al. reported that among those patients who experienced a hospitalized infection while taking anti-TNFα therapy, etanercept and abatacept were associated with the lowest risk of subsequent infection compared to infliximab users [39].

As discussed above, the results on comparative risk of serious infection associated with biological therapies were somewhat inconsistent across studies. Curtis et al. showed that much wider variability in patients’ risk of serious infection or hospitalized bacterial infection was related to demographics, comorbidities, higher prednisolone doses, and other patient-specific risk factors compared with exposure to biological agents [35, 38]. Similar data were reported by the REAL Study conducted in Japan [40]. In addition, RA is known to confer a great risk of infectious disease irrespective of whether biological therapy is used [48]. Such high background rates of infection as a result of these risk factors may confound interpretation of a small difference in the risk of serious infection between treatment groups. In the present study, there was no significant difference in adjusted HRs of overall hospitalized infection between the treatment groups with different biological agents. The patient-specific factors more significantly influenced the risk for hospitalized infection. A recent systemic literature review to inform the task force responsible for the 2016 update of EURLA recommendations has confirmed that RA patients receiving biological therapy have an increased risk of serious infections compared with those on non-biological DMARD therapy, but there are generally no differences in adjusted HRs for serious infections across biological agents [49].

In this study, adalimumab had a significantly higher adjusted HR for pulmonary hospitalized infection compared with tocilizumab. This finding suggested that the contribution of each biological agent to the risk of developing hospitalized infections may be specific to the anatomic site. We found that the rate of pulmonary infection was 80% of all hospitalized infectious events during adalimumab therapy, while this type of infection accounted for 36.8% of all hospitalized infection in patients receiving tocilizumab. Skin and soft tissue infection and gastrointestinal infection accounted for 36.9% of all hospitalized infection in the tocilizumab group, while there were no cases of these types of hospitalized infection in the adalimumab group. The frequent occurrence of such non-pulmonary severe infectious events besides pulmonary infection was observed in previous RCTs, postmarketing surveillance studies, and registry studies on tocilizumab therapy [40, 50–54]. The Japanese REAL registry study showed that an IR of non-pulmonary infection was significantly higher in the tocilizumab group compared with the anti-TNFα agent group [40]. In addition, it has been reported that the antibody type in anti-TNFα agents is associated with an increased risk of granulomatous infection [15]. In this study, *Pneumocystis jirovecii* pneumonia (PCP), tuberculosis, or pulmonary mycosis occurred in patients receiving adalimumab while these infectious events were not observed during tocilizumab therapy, which may also explain the reason for the higher adjusted HR of pulmonary hospitalized infection during adalimumab therapy. Although the implementation status of tuberculosis screening, pneumococcal vaccination, PCP prophylaxis, and counseling about infection prevention should be considered as possible confounding factors, these factors were difficult to measure and control in this real-world retrospective cohort study. Unfortunately, few studies have directly compared adjusted HRs for anatomic site-specific severe infections among various biological agents. Given the relatively lower absolute IRs of hospitalized infection in most previous trials and observational studies, it may be difficult to compare directly hospitalized infections classified by organ between various biological agents.

There are at least two limitations in this study. First, the present study was performed retrospectively, which may confer certain inherent limitations on the study. In this type of study, the data being used is not designed to be used in a study, and therefore, some data may be inaccurate and even unavailable. Additionally, we did not include disease activity values as baseline characteristics because participating hospitals used different disease activity measures: some hospitals used the disease activity score with 28-joint counts (DAS28) and others used the clinical disease activity index (CDAI). Recent studies suggested that higher disease activity was associated with a higher probability of developing infections [55, 56]. Thus, the present study is subject to confounding from RA disease activity of each patient. We included other RA-related features (RA duration, radiological stage, and functional class) in the Cox regression analyses and found that RA class 3/4 was the significant factor associated with the risk of overall hospitalized infections. Second, it was difficult to compare adjusted HRs for infection site-specific (except pulmonary infections) or causative pathogen-specific hospitalized infections between biological treatment groups, because further classification of hospitalized infectious events by involved organs and causative pathogens resulted in a small sample size of events in each treatment group. For such subgroup analyses, it was difficult to obtain sufficient statistical power to detect a difference in HRs after adjustment for confounding factors between various biological agents. Larger-scale observational studies are required to determine, with sufficient statistical power, comparative risks of infection site-specific and causative pathogen-specific hospitalized infections between various biological therapies for RA.

## Conclusions

The magnitude of the risk associated with overall hospitalized infection is not determined by the type of biological agents used to treat RA patients. Rather, patient-specific factors, in particular age, RA function class 3/4, BMI <18.5, prednisolone use (≥7.5 mg/day), and chronic lung disease, were significantly associated with increased risks of overall hospitalized infection. For pulmonary infections requiring hospitalization, the risk was significantly higher in RA patients receiving adalimumab compared with tocilizumab. Patient-specific factors, especially BMI <18.5, prednisolone use (≥7.5 mg/day), diabetes mellitus, and chronic lung disease, also contributed to developing pulmonary infection diseases during biological therapy for RA. Patient-specific risk factors should be more carefully considered and, if possible, modified in RA patients who are scheduled to receive, and who are receiving, biological therapy.
